# Supplementary material for: Prefrontal cortex state representations shape human credit assignment
Source: eLife. 2023 Jul 3;12:e84888. doi: 10.7554/eLife.84888 (PMC10351919; doi:10.7554/eLife.84888)
Supplement: Supplementary file 1. — (a) Choice Phase ROI coordinates. (b) Feedback Phase ROI coordinates. (c) Conjunction ROI coordinates from cross-timepoint analysis. (d) Parametric modulation ROI coordinates. (e) List of RL Models included in model comparison, including their respective free parameters indicated with the ×. V denotes valenced terms for either the learning rate (LR) or credit assignment (CA) parameters in the model. (f) Logistic RL algorithm parameters indicating model behavior at upper and lower bounds. (g) Mean AIC and SE for each model. Mean AIC for V-LR, V-CA was the max in the set. Mean values are plotted below in Figure 3—figure supplement 2. (h) Model Comparison. Model Comparison was performed by minimizing Δ AIC, which was computed as the difference between each participants best-fitting model and each model in the set (see Methods). Δ AIC was the lowest for the V-LR, V-CA model indicating that the model captured the behavioral data better than other models in the set, and in instances in which a participant’s data was better fit by another model the V-LR, V-CA model could explain the data equally as well. Δ AIC with individual points is plotted below in Figure 3—figure supplement 2. [file elife-84888-supp1.docx]

| Region | *Peak MNI Coordinates* | | | *Z_peak_* | *k* |
| --- | --- | --- | --- | --- | --- |
|  | **x** | **y** | **z** |  | |
| Parietal R | 46 | -54 | 52 | 5.36 | 331 |
| OFC M | -4 | 36 | -24 | 4.02 | 81 |
| dlPFC R | 40 | 52 | 26 | 4.81 | 254 |
| mPFC | 2 | 54 | -2 | 5.56 | 321 |
| Parietal L | -58 | -50 | 36 | 4.78 | 292 |
| ACC | -2 | 10 | 42 | 5.14 | 291 |
| OFC R | 42 | 52 | 6 | 4.70 | 260 |
| OFC L | -40 | 40 | -4 | 4.71 | 253 |
| dlPFC L | -42 | 8 | 40 | 4.36 | 267 |

***Supplementary File 1a.***

| Region | *Peak MNI Coordinates* | | | *Z_peak_* | *k* |
| --- | --- | --- | --- | --- | --- |
|  | **x** | **y** | **z** |  |  |
| Parietal R | 48 | -38 | 54 | 6.45 | 388 |
| mPFC | 0 | 52 | 22 | 5.32 | 388 |
| dlPFC R | 42 | 28 | 28 | 5.50 | 389 |
| Parietal L | -38 | -38 | 54 | 5.16 | 377 |
| OFC R | 26 | 56 | -10 | 4.72 | 238 |
| dlPFC L | -40 | 20 | 32 | 5.57 | 327 |
| Hippocampus R | 40 | -18 | -10 | 4.89 | 213 |
| OFC L | -48 | 46 | -4 | 5.18 | 275 |
| ACC | -2 | 24 | 32 | 4.75 | 330 |
| OFC M | 0 | 46 | -20 | 4.33 | 142 |

***Supplementary File 1b.***

| Region | *Peak MNI Coordinates* | | | *Z_peak_* | *k* |
| --- | --- | --- | --- | --- | --- |
|  | **x** | **y** | **z** |  |  |
| OFC R | 32 | 46 | -10 | 5.26 | 106 |
| mPFC | 6 | 50 | -2 | 5.94 | 158 |

***Supplementary File 1c.***

| Region | *Peak MNI Coordinates* | | | *Z_peak_* | *k* |
| --- | --- | --- | --- | --- | --- |
|  | **x** | **y** | **z** |  |  |
| Ventral Striatum L | -10 | 6 | -10 | 5.64 | 140 |
| Ventral Striatum R | 8 | 16 | -12 | 5.33 | 112 |
| vmPFC | 4 | 56 | -8 | 4.99 | 27 |

***Supplementary File 1d.***

| **Model No.** | **Model** | **bias** | | **m** | **prior** | **a** | **a_pos_** | **a_neg_** | **decay** | **CA** | **CA_pos_** | **CA_neg_** | **No. params** |
| --- | --- | --- | --- | --- | --- | --- | --- | --- | --- | --- | --- | --- | --- |
| 1 | Baseline | | $\times$ | $\times$ |  | $\times$ |  |  |  |  |  |  | 3 |
| 2 | Decay | | $\times$ | $\times$ | $\times$ | $\times$ |  |  | $\times$ |  |  |  | 5 |
| 3 | V-LR, Decay | | $\times$ | $\times$ | $\times$ |  | $\times$ | $\times$ | $\times$ |  |  |  | 6 |
| 4 | V-LR, CA | | $\times$ | $\times$ | $\times$ |  | $\times$ | $\times$ |  | $\times$ |  |  | 6 |
| 5 | V-CA | | $\times$ | $\times$ | $\times$ | $\times$ |  |  |  |  | $\times$ | $\times$ | 6 |
| 6 | V-CA, V-LR | | $\times$ | $\times$ | $\times$ |  | $\times$ | $\times$ |  |  | $\times$ | $\times$ | 7 |

***Supplementary File 1e.***

| **Model Parameter** | **Parameter Description** | **Upper-bound** | **Lower-bound** |
| --- | --- | --- | --- |
| bias | Baseline investment tendencies. | 2.0 – Baseline tendency to invest full amount. | 0 – Baseline tendency to invest minimal amount. |
| m | Slope of logistic function associating V_t_ and predicted investments. | 2.0 – Little evidence needed to shift investment strategies. | 0.2 – More evidence required before shifting strategies. |
| prior | Initial value of V on trial 1 before observing any outcomes. | 2.0 –Invest maximally on trial 1. | 0 – Invest minimally on trial 1. |
| a | Learning rate quantifying degree of update to state  V_t_ from PE term. | 1.0 – PEs maximally updated state  V_t_. | 0 – No influence of PEs on state V_t_ |
| a_pos_ | Learning rate for gains. | 1.0 – PEs > 0 maximally updated state V_t_. | 0 – PEs > 0 did not update state V_t_. |
| a_neg_ | Learning rate for losses. | 1.0 – PEs < 0 maximally updated state V_t_. | 0 – PEs < 0 did not update state V_t_. |
| CA | Credit assignment parameter capturing spread of credit across states. | 1.0 – PEs only update V_t_ of relevant state. | 0 – PEs update  V_t_ of all states. |
| CA_pos_ | Credit assignment for gains. | 1.0 – PEs > 0 only update V_t_ of the relevant state. | 0 – PEs > 0 update V_t_ of all states. |
| CA_neg_ | Credit assignment for losses. | 1.0 – PEs < 0 only update V_t_ of the relevant state. | 0 – PEs < 0 update V_t_ of all states. |
| decay | Degree of forgetting | 1.0 – Complete decay of V_t_. | 0 – No forgetting. |

***Supplementary File 1f.***

| **Model No** | **Model** | **Task** | **Mean AIC** | **SE** |
| --- | --- | --- | --- | --- |
| 1 | Baseline | Social  Bandit | -324.93  -349.11 | 30.84  21.00 |
| 2 | Decay | Social  Bandit | -232.06  -285.99 | 19.51  17.75 |
| 3 | V-LR, Decay | Social  Bandit | -203.83  -277.41 | 16.68  18.55 |
| 4 | V-LR, CA | Social  Bandit | -202.68  -259.47 | 17.00  17.30 |
| 5 | V-CA | Social  Bandit | -208.54  -273.25 | 16.75  17.93 |
| 6 | V-LR, V-CA | Social  Bandit | -200.18  -258.25 | 16.65  16.99 |

***Supplementary File 1g.***

| **Model No** | **Model** | **Task** | **Δ AIC** | **SE** |
| --- | --- | --- | --- | --- |
| 1 | Baseline | Social  Bandit | 127.66  92.85 | 25.17  14.98 |
| 2 | Decay | Social  Bandit | 34.79  29.73 | 9.35  5.28 |
| 3 | V-LR, Decay | Social  Bandit | 6.56  21.16 | 4.77  5.23 |
| 4 | V-LR, CA | Social  Bandit | 5.40  3.32 | 4.94  4.79 |
| 5 | V-CA | Social  Bandit | 11.26  16.99 | 3.32  5.71 |
| 6 | V-LR, V-CA | Social  Bandit | 2.91  2.00 | 4.88  4.56 |

***Supplementary File 1h****.*
